# Aortic Periannular Abscess Missed by Transthoracic Echocardiography: A Case Report

**DOI:** 10.1002/ccr3.70114

**Published:** 2025-01-16

**Authors:** Wenjuan He, Yudong Peng, Yan Yi, Han Hu, Jinzhi Lu, Keming Chen, Qingsong Zeng

**Affiliations:** ^1^ Department of Obstetrics and Gynecology The First Affiliated Hospital of Yangtze University Jingzhou China; ^2^ Department of Ultrasound The First Affiliated Hospital of Yangtze University Jingzhou China; ^3^ Department of Ophthalmology Jingmen Cental Hospital, Jingmen Cental Hospital Affiliated to Jingchu University of Technology Jingmen China; ^4^ Department of Laboratory Medicine, Hubei Clinical Medicine Research Center for Individualized Cancer Diagnosis and Therapy The First Affiliated Hospital of Yangtze University Jingzhou China

**Keywords:** aortic periannular abscess, case report, infective endocarditis, small vegetation, transesophageal echocardiography, transthoracic echocardiography

## Abstract

An aortic periannular abscess (PA) is a critical consequence of infective endocarditis (IE). In our case report, the patient's clinical symptoms were only fever, cough, and shortness of breath. He was then diagnosed with aortic PA, which was overlooked in the initial TTE assessment but later identified through transesophageal echocardiography (TEE). The patient underwent surgery immediately and recovered well. This case underscores the importance of TEE in revealing small, yet critical, intracardiac lesions that may be missed by TTE.

**Summary**

- In optimal imaging conditions, transthoracic echocardiography (TTE) can detect vegetations as small as 2 mm on valves.
- However, in our case, the 2‐mm vegetation was overlooked by TTE.

## 1 Introduction

Without aggressive treatment, the mortality rate of endocarditis is almost 100%, and even with the best treatment, one‐third of patients still die [1]. An aortic periannular abscess (PA), also referred to as an aortic root abscess, is a significant and frequently encountered complication associated with infective endocarditis (IE). Research indicates that approximately 22% of individuals with aortic valve disease are found to have PA [2]. Diagnostic approaches to IE are varied and encompass a range of imaging techniques such as transthoracic echocardiography (TTE), transesophageal echocardiography (TEE), multidetector computed tomography (CT), cardiac magnetic resonance imaging (MRI), and nuclear imaging. While advanced imaging options like MRI and nuclear imaging are accessible primarily at major tertiary healthcare facilities, echocardiography remains the predominant diagnostic tool in cardiac centers. It is frequently the first imaging method of choice in the evaluation and management of IE due to its widespread availability and diagnostic efficacy [3]. In the realm of diagnostic cardiology, TTE emerges as the quintessential modality for initial assessment, while TEE is reserved for select scenarios. TTE can serve as the preferred imaging modality for suspected cases of IE. For patients with clinically suspected IE but negative or inconclusive TTE results, TEE is recommended. In patients with clinically suspected IE who have artificial heart valves or intracardiac devices, TEE is advised. Cardiac CT is more accurate than TEE in diagnosing perivalvular and periprosthetic complications of infective endocarditis, such as abscesses, pseudoaneurysms, and fistulas. In patients where IE has been ruled out, or even in those with suspected IE, a whole‐body CT can provide an alternative diagnosis as it aids in identifying other foci of infection [4]. A meta‐analysis has demonstrated that, compared to the gold standard of surgical inspection or autopsy assessment [5], the misdiagnosis rates for vegetations and circumferential extension by TEE are 14% and 18%, respectively. The majority of cases where TEE failed to detect vegetations or circumferential extension involve prosthetic mechanical aortic valves. In an ideal imaging environment, the sensitivity of TTE is such that it can identify even the most minute vegetations, as diminutive as 2 mm in size, on cardiac valves [6]. However, in the case under discussion, the TTE examination regrettably failed to detect a vegetation of precisely 2 mm, highlighting the potential limitations of this modality under certain clinical conditions.

Here we report on a 27‐year‐old male who presented with fever, cough, and dyspnea. A small abscess was not detected by transthoracic echocardiography, and the diagnosis was later confirmed by transesophageal echocardiography.

## 2 Case Presentation

A 27‐year‐old male, with no significant past medical history, arrived at the emergency department reporting persistent fever, cough, and shortness of breath for the preceding week. He has no history of smoking or alcohol consumption. He was given oral antibiotics (cefuroxime) for 5 days, but the treatment was ineffective and the cause of the fever still could not be diagnosed.

## 3 Diagnostic Assessment

**FIGURE 1:**
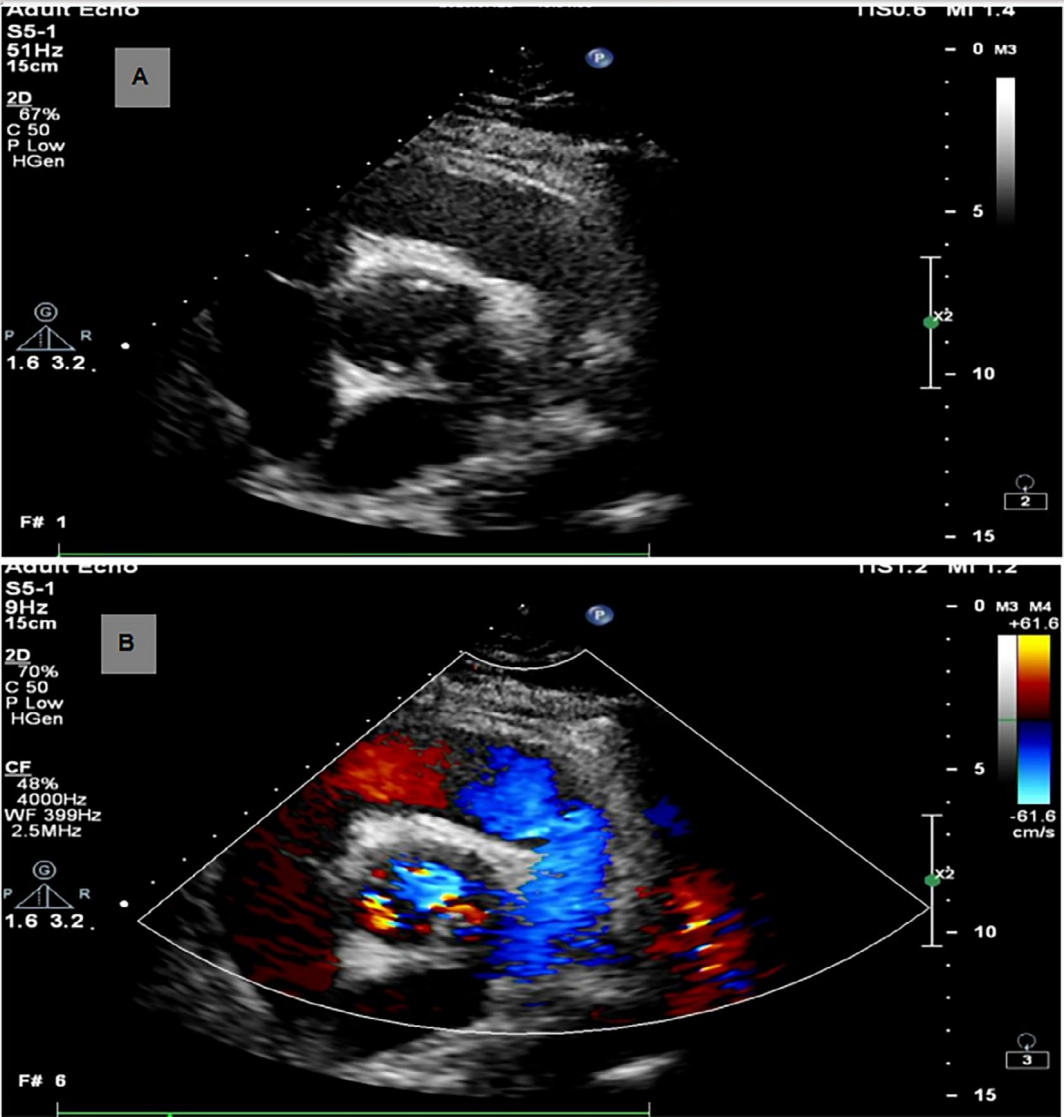
Transthoracic echocardiography imaging. (A) Aortic valve short axis. (B) Aortic valve short axis with color‐flow Doppler.

**FIGURE 2:**
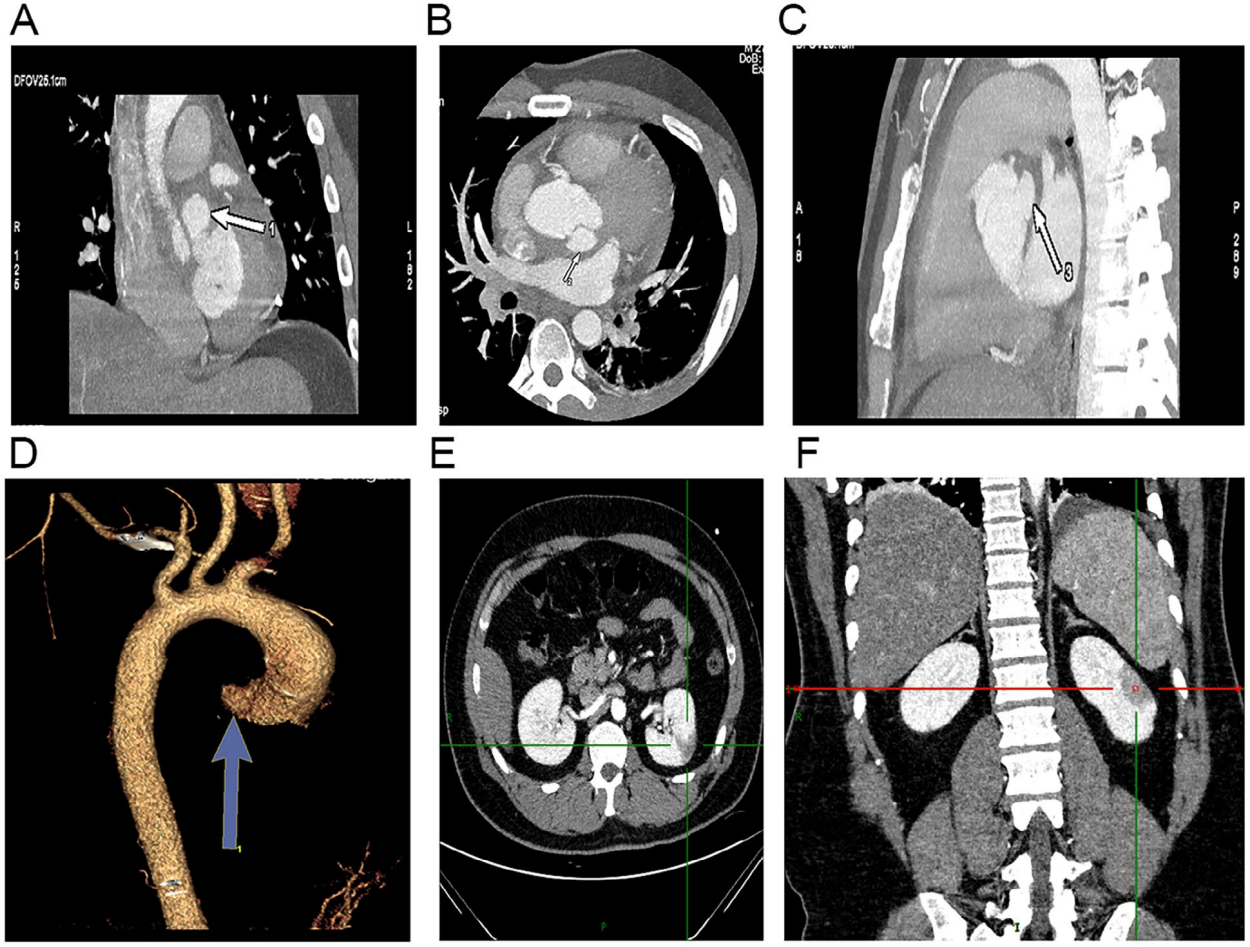
The images of the cardiac CT. (A) The coronal view of the heart, arrow points to the aneurysm of the aortic sinus. (B) The transverse section of the heart. (C) The sagittal view of the heart. (D) 3D image. (E) The transverse section of the kidney, the line indicates the area of renal infarction. (F) The coronal section of the kidney.

**FIGURE 3:**
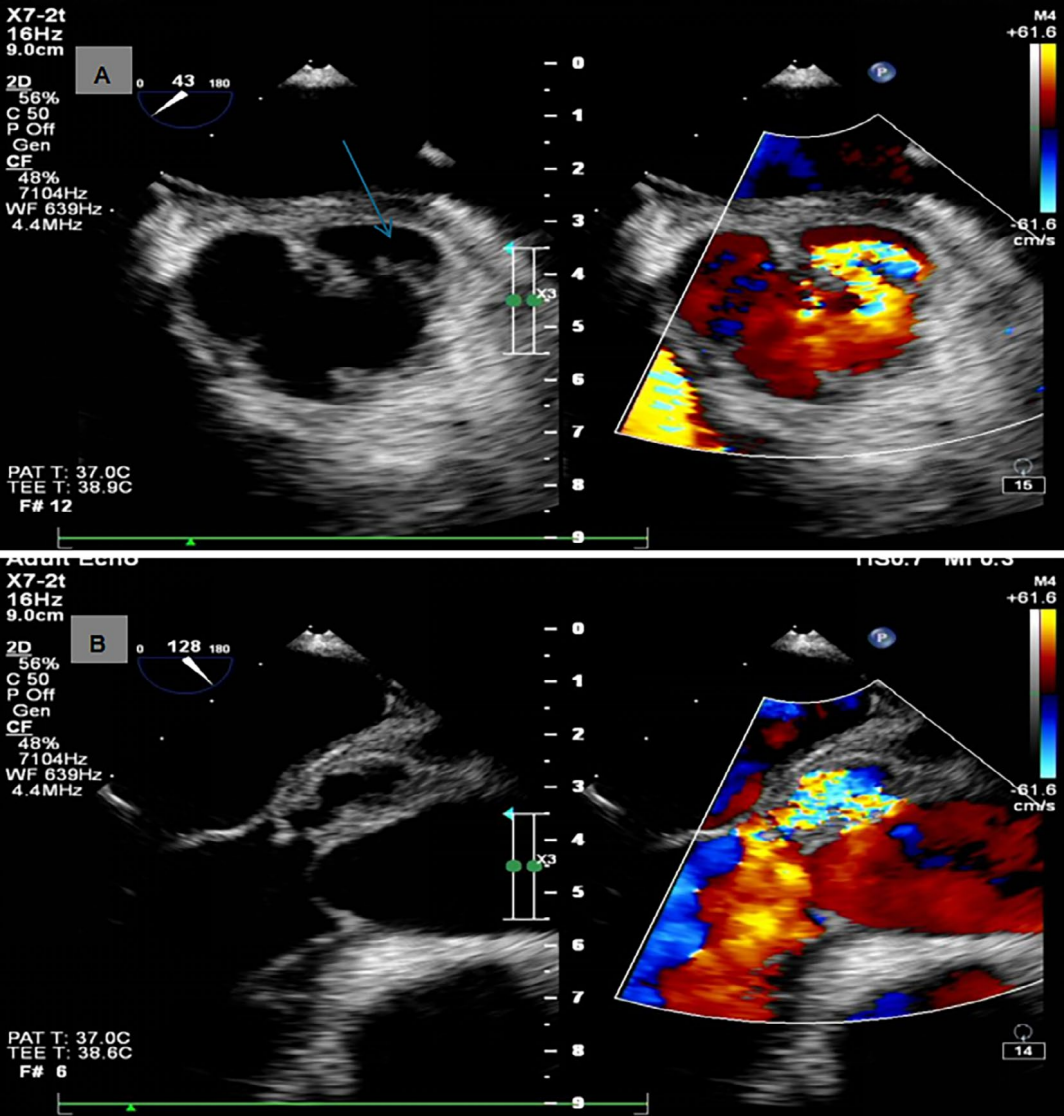
Transesophageal echocardiography imaging. (A) Aortic valve short‐axis with color‐flow Doppler, arrowhead points to periannular abscess. (B) Aortic valve long‐axis with color‐flow Doppler.

Blood tests were immediately conducted upon admission to the hospital. Blood routine showed leukocytes 18.11 × 10^9^ (normal range 4–10 × 10^9^/L), neutrophils absolute value 13.86 × 10^9^ (normal range 2–7 × 10^9^/L), and C‐reactive protein 157.29 (normal range 0–5 mg/L). This indicates the presence of an infection. Renal function showed uric acid 525.8 (normal range 149–416 μmol/L). Liver function showed no significant abnormality. Auscultation revealed no heart murmurs or abnormal breathing sounds. An electrocardiogram (ECG) was performed 1 h after admission. The ECG did not reveal any abnormalities. A TTE was performed 2 h after admission. TTE suggested pericardial effusion, abnormal aortic valve development, and no significant abnormalities in left heart function. (Figure 1). A chest CT scan was performed 3 h after admission. Chest CT revealed enlargement of the left ventricle, perfusion around the ascending aorta, and pericardial effusion. Pericardiocentesis was performed under ultrasound guidance, draining a pale yellow fluid, no pathogens were cultured, but the patient continued to have recurrent fever with temperatures fluctuating between 38°C and 39°C. No other sites of infection were found, and the cause of fever remained undiagnosed. A cardiac CT scan was performed 4 h after admission. CT angiography suggested significant dilation of the ascending aortic sinus, with an abnormal protruding sac‐like high‐density image adjacent to the left coronary sinus, suspected to be an aortic sinus aneurysm (Figure 2). A bilateral CT scan was conducted 5 h after admission. Bilateral renal CT showed multiple small infarcts in both kidneys. Peripheral vascular damage was observed, with suspected aortic valve congenital abnormality and possible aortic sinus aneurysm, leading to a high suspicion of IE. Within 24 h after hospital admission, a TEE was performed, revealing a sac‐like echo in the aortic sinus, measuring 19 × 15 × 23 mm in size. There were multiple communication orifices visible with the aortic sinus, one of which had an internal diameter of 4 mm, and it was also connected to the left ventricular outflow tract, with an internal diameter of 7 mm. Within the sac‐like echo, multiple band‐like echoes were observed floating, one of which measured 7 × 2 mm, considered to be a vegetation. This is consistent with the appearance of a PA of the aortic valve, and the floating vegetation within the lumen has the potential to detach at any time (Figure 3).

**FIGURE 4:**
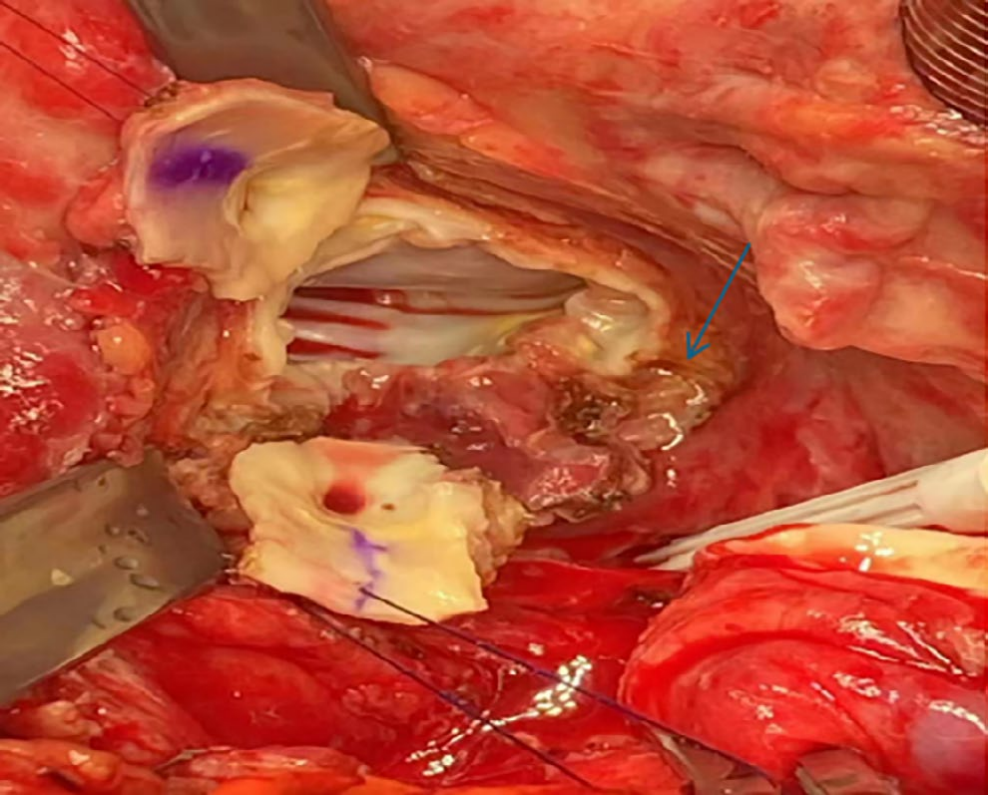
Intraoperative images showing periannular abscess during aortic exploration, arrowhead points to periannular abscess.

According to the Duke diagnostic criteria [4], the diagnosis of infective endocarditis in this patient is unequivocal. The diagnosis of a prosthetic aortic valve for this patient is indisputable. The patient underwent immediate surgical intervention, including debridement of infected tissue, aortic valve with ascending aorta replacement, and coronary artery transplantation (Figure 4).

## 4 Follow‐Up

Following a 1‐day period of intensive care unit (ICU) hospitalization, the patient was subsequently transferred to a standard ward for continued medical management. The patient experienced a postoperative fever for three consecutive days and was treated with a combination of ceftizoxime sodium and vancomycin. The patient was discharged after a 9‐week hospitalization period. The patient underwent a TTE for follow‐up 1 month later, and no significant abnormalities were observed. The patient was very satisfied with the timely diagnosis and treatment.

## 5 Discussion

This case emphasizes that small aortic PA cannot be detected by TTE in the case of IE. Most aortic PA can be diagnosed by TTE, but smaller ones are prone to being missed and require TEE for diagnosis. Compared with TEE, TTE has lower sensitivity in detecting vegetations, especially smaller ones [3]. Data pertaining to the sensitivity of abscess detection are as follows: the TTE exhibits a sensitivity ranging from 28% to 36%, while the TEE demonstrates a significantly higher sensitivity, with a range of 80%–100%. Correspondingly, the specificity for TTE and TEE is 99% and 95%, respectively [7]. TEE has a higher resolution and can provide a more thorough examination of the aortic valve and surrounding structures. The detection of PA is crucial because it often requires surgical intervention to prevent further complications, including valve dysfunction, fistula formation, abscess rupture, and embolism. Ultrasound technicians should carefully examine the valves impacted by high‐speed blood flow for the presence of vegetations, and when TTE results are negative, further TEE should be performed to confirm the diagnosis.

## 6 Conclusion

Vegetations are a necessary condition for the diagnosis of IE, and TEE is more sensitive in observing perivalvular vegetations, making it an indispensable tool for assessing patients with suspected IE. When IE is highly suspected clinically, and TTE is negative or PA is suspected, TEE can be performed, combined with the patient's clinical manifestations to confirm the diagnosis. Timely recognition and intervention can significantly improve patient prognosis.

## Author Contributions

**Wenjuan He:** conceptualization, writing – original draft. **Yudong Peng:** conceptualization, writing – review and editing. **Yan Yi:** data curation, resources. **Han Hu:** writing – review and editing. **Jinzhi Lu:** writing – review and editing. **Keming Chen:** data curation, investigation, visualization. **Qingsong Zeng:** funding acquisition, visualization.

## Ethics Statement

The study was approved by the Ethics Committee of The First Affiliated Hospital of Yangtze University.

## Consent

The authors have secured written informed consent from the patients, which is a prerequisite for the dissemination of this case report, including any associated data and visual materials.

## Conflicts of Interest

The authors declare no conflicts of interest.

## Data Availability

In the spirit of transparency and scientific collaboration, all contributing authors concur on the commitment to ensure that all materials delineated within our manuscript are made readily accessible. This includes the full complement of pertinent raw data, which will be furnished to the broader research community for the purpose of unrestricted reuse and reanalysis.
